# Association of TLR7 and TLR9 genes polymorphisms in Egyptian patients with systemic lupus erythematosus

**DOI:** 10.1016/j.heliyon.2022.e11680

**Published:** 2022-11-21

**Authors:** Marwa M. Azab, Fatma M. Mostafa, Mayada Khalil, Mona Salama, Ali A. Abdelrahman, Aya A. Ali

**Affiliations:** a Department of Microbiology and Immunology, Faculty of Pharmacy, Suez Canal University, Ismailia, Egypt; b Department of Rheumatology and Rehabilitation, Faculty of Medicine, Benha University, Benha, Egypt; c Department of Clinical Pathology, Faculty of Medicine, Port Said University, Port Said, Egypt; d Department of Microbiology and Immunology, Faculty of Pharmacy, Sinai University, Arish, Egypt

**Keywords:** TLR7, TLR9, Systemic lupus erythematosus

## Abstract

**Introduction:**

Systemic lupus erythematosus (SLE) is a chronic, inflammatory, multiorgan, systemic autoimmune disease. It is characterized by the high production of autoantibodies against nuclear compounds. TLRs (toll-like receptors 7/9) are pattern-recognition receptors that recognize nucleic acids and induce proinflammatory responses by activating NF-kB and producing type I interferon, which play a role in eliciting innate/adaptive immune responses and developing chronic inflammation. TLR7 and TLR9 single nucleotide polymorphisms (SNPs) have been linked to systemic lupus erythematosus in numerous studies (SLE). In this work, we wanted to evaluate and analyze single nucleotide polymorphisms (SNPs) in the TLR7 (rs3853839) and TLR9 (rs187084) genes among Egyptian SLE patients and healthy controls.

**Method:**

Whole blood samples were taken from 100 SLE patients and 100 controls; DNA was extracted and then processed for TLR7 rs3853839 and TLR9 rs187084 single nucleotide polymorphisms analysis by real-time polymerase chain reaction technology and restriction fragment-length polymorphism. We also assessed the association between TLR 7 and TLR 9 genes polymorphism with SLE clinical parameters.

**Results:**

Our results showed that TLR7 rs3853839 CG genotypes and G allele were significantly associated with SLE. Also, TLR7 rs3853839 genotypes and alleles were significantly associated with nephritis, arthritis, oral ulcers, and thrombocytopenia.

Whereas genotypes and alleles of TLR9 were not significantly associated with the risk nor the clinical characteristics of SLE except for malar rash.

**Conclusion:**

In the investigated Egyptian cohort, our findings suggest that TLR7 rs3853839 gene polymorphisms increase the risk for SLE development and play a role in developing clinical characteristics, especially nephritis.

## 1 Introduction

SLE is a worldwide chronic multisystemic autoimmune illness with various clinical symptoms, laboratory and immunological abnormalities, and variable outcomes, course, and complications (Pons-Estel et al., 2017; Fava and Petri 2019; Thanou et al., 2021). Patients show various clinical symptoms; cutaneous lesions are present in more than 80% of cases and are characterized by: a distinctive butterfly rash across the cheeks, which relapses, and chronic discoid lesions. One or more subsequent manifestations may occur: fatigue, painful joints, nephritis, pleuritis, pericarditis, CNS abnormalities, accelerated atherosclerosis, and severe renal disease (Marshak-Rothstein, 2006).

B cells play a crucial role in autoimmune disorders, which are frequently characterized by certain autoantibody patterns and include a lack of B cell tolerance. Systemic lupus erythematosus is a prototypic disease linked to B cell hyperactivity (SLE). TLRs, which detect nucleic acids in endosomes, regulate the loss of B cell tolerance to autoantigens in SLE patients in a cell-intrinsic manner. The extrafollicular B cell response and the germinal centre reaction, which are important in the formation of autoantibodies and disease pathogenesis, are driven by TLR7 (Fillatreau et al., 2021).

TLR arbitrated signaling pathways persuade a proinflammatory micro-environment to eliminate pathogens. Though, in SLE, TLRs may enhance chronic inflammatory reactions (Aranda-Uribe et al., 2021).

TLRs are considered the core players of SLE pathogenesis (Guggino et al., 2012). Many researchers have confirmed that TLRs genes polymorphism plays an essential role in developing this disease (Devaraju et al., 2015). TLR7 gene is found in X chromosome Xp22.2, which encodes TLR7 protein (Banchereau and Pascual, 2006). TLR7 is an intracellular pattern recognition receptor (PRR) that identifies uridine (U) and guanosine (G)-rich single-stranded RNA (Li et al., 2012). Immune cells having TLR7-null mutation exhibit severe impairments regarding ssRNA sensing and discharge inflammation mediators, including IFN-γ, IFN-α, and TNF (Banchereau and Pascual 2006, Mukherjee et al., 2019). Targeting TLR in SLE may be therapeutically advantageous based on the roles of TLR7 and TLR9 in the effector function of B cells in lupus-like disease and SLE patients as well as the distinctive characteristics of TLR signaling in B cells (Fillatreau et al., 2021).

SNPs are resulted from mutations that generate base-pair differences amongst chromosome sequences (Leaché and Oaks, 2017). Recent advancements in human genomics have led to experimental evidence on many polymorphism residues in TLR proteins and genes. The immunological responses can be impacted by these TLR polymorphisms (Mukherjee1 et al., 2019).

The relation between TLR7 SNPs and SLE can be illustrated by the overproduction of proinflammatory cytokines and type I IFN (Savitsky et al., 2010). Genetic research about SLE confirms an association between copy number variations (CNV) and SNP within TLR7 gene locus and the susceptibility of SLE (Lau et al., 2005; Shen et al., 2010; Kawasaki et al., 2011; Wang et al., 2014; Ortiz 2019). The SNP of TLR7 rs3853839 C/G, situated in the 3′ untranslated region (UTR) of TLR7, has been linked with the increase in TLR7 mRNA and the expression of TLR7 protein and up-regulation of IFN stimulated genes (ISGs) (Shen et al., 2010; Wang et al., 2014).

It is obvious now that miRNAs work to regulate TLR signaling via targeting the expression process or via modifying cytokines, adaptor molecules, and regulators downstream (O'neill et al., 2011). TLR7 rs3853839 (G/C) can affect the binding of miRNAs and accordingly TLR7 expression and/or responsiveness (Raafat et al., 2018). TLR7 transcripts are amplified in G-allele carriers, and they are more predictable to encompass anti-RNA-associated autoantibodies than C-allele carriers (Shen et al., 2010).

The TLR-9 gene is found in chromosome 3p21.3, one of the SLE susceptibility regions (Elloumi et al., 2017). The rs187084, situated in the promoter section, can affect the expression of TLR-9 and influence the production of autoantibodies (Hamann et al., 2006). Furthermore, a meta-analysis study and a systematic review claimed that the development of SLE in Asian populations was linked with TLR7 and TLR9 polymorphisms (Lee et al., 2012). TLR9 SNPs modify immune responses via interfering with the receptor binding activity to the ligand, which leads to signal defects and causes cytokine secretion impairment from immune cells (Mukherjee et al., 2019). It is known that the TLR-9 receptors are stimulated through hypomethylated cytosine–phosphate–guanosine (CpG) DNA and can modulate and/or initiate autoimmunity via provoking inflammatory cells and producing antibodies or cytokines (Akira and Takeda, 2004; Yang et al., 2012).

Functional and Genetic research developed proof that supports that SNPs of the TLR9 gene may be allied with the susceptibility to and the severity of SLE, however, this subject needs further research (Elloumi et al., 2017).

## 2 Materials and methods

### 2.1 Patients

Our work was carried out in two groups:

Group 1: 100 Egyptian SLE patients from different university hospitals, the age of the patient group ranged from 18 to 51 years old, with a mean age of 33.97 (SD = 12.97) years. Eight (8%) of our cases were males, while 92 (92%) were females with a mean disease duration of 6 years.
Group 2: 100 healthy Egyptian individuals as control, who were age and gender-matched to our studied patients.

Our inclusion criteria were males, and females >18 diagnosed SLE patients based on American College of Rheumatology (ACR) diagnostic criteria.

Exclusion criteria were patients with malignancy, other autoimmune diseases, or other chronic conditions (diabetes mellitus). The control group was selected with matched age and sex.

Since there is no universal agreement on what constitutes remission, low disease activity (LDA), or how to treat SLE over the long term, the therapy of SLE is highly varied. These limitations might result in inadequate therapeutic approaches. In our study, all of our patients passed through different stages of disease activity and have been treated with different regimens. The choice and timing of drug administration and tapering until withdrawal is highly varied according to the symptoms and it was performed under physician supervision. All of our patients have been treated with almost all SLE therapies (*Mycophenolate mofetil*, Methotrexate, Cyclophosphamide, Azathioprine, and Corticosteroids) at different stages of disease activity.

### 2.2 Ethics statement

The patients were selected from rheumatology & immunology clinics, at Suez Canal University Hospital. All patients who took part in the study gave their informed written consent. Ethical approval for the study was obtained from the Suez Canal Faculty of Pharmacy ethical committee.

### 2.3 Genotyping for the TLR7 rs3853839 and TLR9 rs187084 single nucleotide polymorphisms

#### 2.3.1 DNA extraction

Frozen blood samples were allowed to thaw and used for DNA extraction by QIAamp DNA Mini and Blood Mini kit (QIAGEN GmbH Germany, Düsseldorf, Germany) (Catalog no. 51104, 51106, 51304, 51306) according to the manufacturer's protocol.

#### 2.3.2 Measurement of DNA concentration in the samples

The DNA concentration in each sample was performed using a Nanodrop One spectrophotometer (Thermoscientific, USA) using 1 μl of the sample. DNA samples were stored at −80 °C until further processing.

#### 2.3.3 SNP genotyping

##### 2.3.3.1 Genotyping of TLR7 rs3853839 SNP

Genotyping was performed using the TLR7 rs3853839 TaqMan Genotyping Master Mix assays (ID: C___2259573_10, Catalog # 4351379) (Thermo fisher scientific, USA). The SNP was recognized using the real-time polymerase chain reaction (RT-PCR) protocol with TaqMan Genotyping assays. The PCR was conducted using a reaction volume of 25 μL, including 12.5 μL TaqMan genotyping master mix, No AmpErase UNG (2×), 1.25 μL TaqMan SNP genotyping assay mix, and 20 ng genomic DNA diluted with DNase-RNase free water to 11.25 μL. After that, StepOne™ real-time PCR system (Applied Biosystems, Foster City, CA, USA) was used for the amplification, under the next conditions: initially, the holding step of 95 °C for 10 min, then 40 cycles of 95 °C for 15 s and finally 60 °C for 1 min. The allelic discrimination was performed by SDS software version 1.3.1 (Applied Biosystems). Genotyping was repeated on 10% of the samples at random to ensure repeatability, and the outcomes were 100% consistent.

##### 2.3.3.2 Genotyping of TLR9-rs187084 SNP

TLR9 single nucleotide polymorphism for rs187084 was performed by polymerase chain reaction-restriction fragment length polymorphism (PCR/RFLP) technique, using forward primer: CCT GCC TGC CAT GAT ACC AC and reverse primer: TGC TAG CAC ACC GGA TCA TT at Chromosome 3, promoter region. PCR products were digested by using AflII restriction then, they were fractionated on 2% agarose gel. The detected patterns of polymorphism for TLR9-rs187084 were 242 bp and 79 bp for the T allele and 321 bp for the C allele. Moreover, 10% of the samples were chosen randomly to perform re-genotyping with 100% concordance with the results.

### 2.4 Statistical analysis

Statistical Package for Social Science (IBM SPSS Statistics for Windows, Version 26.0.) was used to analyze our data.

Mean, and standard deviation (SD) were utilized for parametric numerical data, while for non-parametric numerical data, median and range were employed. Frequencies and percentages were employed for non-numerical data. The statistical significance of the difference between the two study group means was determined using the Student T-Test. One-way analysis of variance (ANOVA) was performed to compare the means of the three groups, followed by the Tukey test. The Mann-Whitney test was used to determine the statistical significance of the difference between two research groups in a non-parametric variable. The statistical significance of the difference between more than two study group ordinal variables was determined using the Kruskal-Wallis test. The association between two qualitative variables was investigated using the Chi-Square test. The Chi-squared test was used to determine deviations from Hardy–Weinberg equilibrium expectations. The 95% confidence interval and odds ratio were determined. Statistical significance was defined as a p-value of ≤0.05.

## 3 Results

We studied the possible role of TLR7 rs3853839 and TLR9 rs187084 SNPs in SLE development. The current study was performed on 100 SLE cases, and the Demographic characteristics of healthy studied controls and SLE patients were studied. Our results revealed that the mean age of SLE patients was 33.97 (SD = 12.97) years. Eight (8%) of our cases were males while 92 (92%) of our cases were females. The control group was chosen to correspond in gender and age.

In our study, the median of SLE disease duration was 6 years and ranged from 1 to 11 years, while the SLEDAI score median was 10, ranging from 2 up to 20. Upon Comparing TLR7 rs3853839 and TLR9 rs187084 genotypes and alleles with disease duration and SLEDAI, the only significant association was detected between the genotypic distribution of TLR7 rs3853839 and SLEDAI score, where the P-value was 0.003.

**Figure 1:**
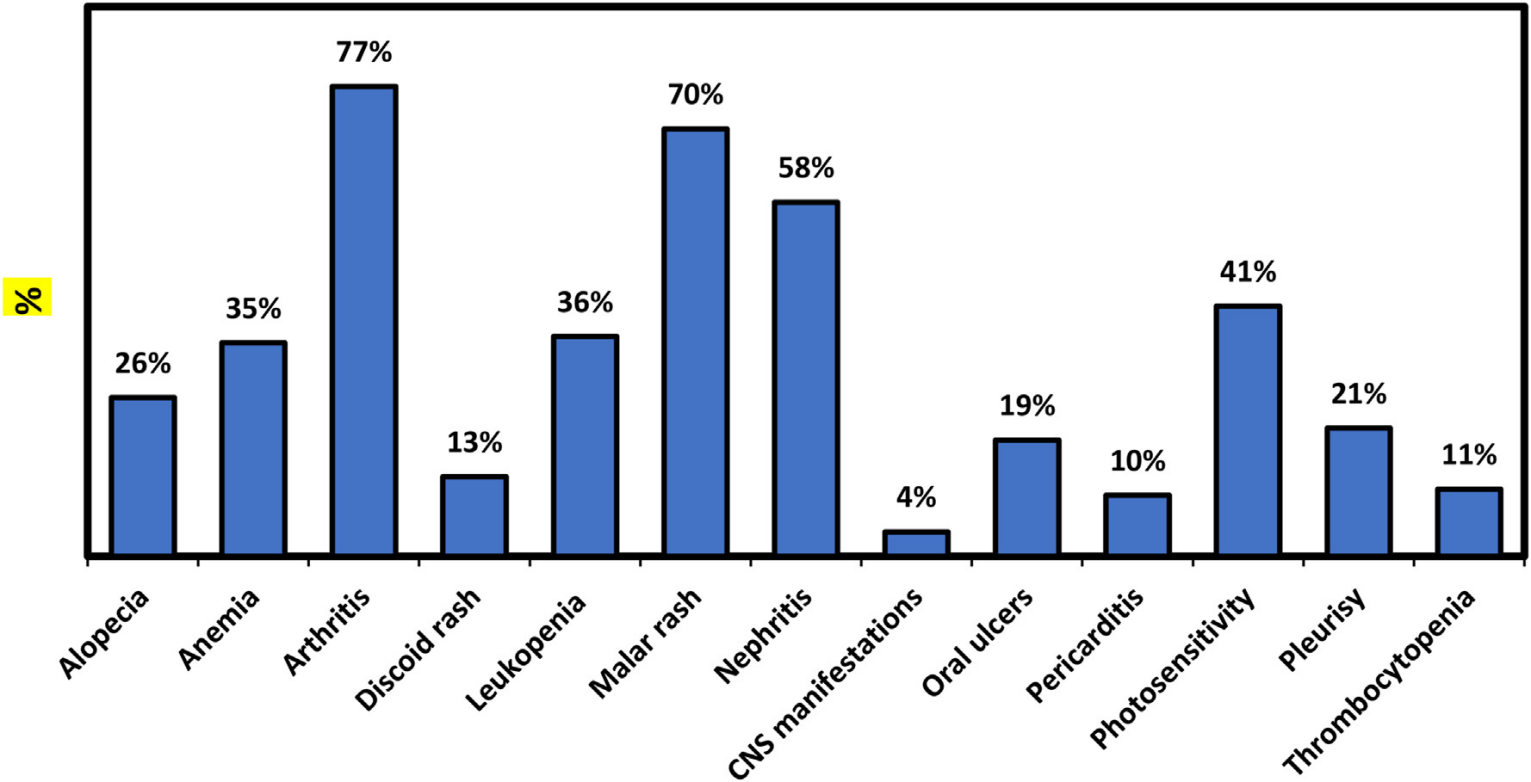
Clinical characteristics of SLE patients. Arthritis affected 77% of patients, followed by Malar rash, which affected 70%, and nephritis, which affected 58%. Only 4% of patients acquired CNS signs. Alopecia was detected in 26% of patients, 35% of patients had anemia, 13% of patients had discoid rash, 36% of patients had leukopenia, 19% of patients had oral ulcers, 10% of patients had pericarditis, 41% of patients had photosensitivity, 21% of patients had pleurisy, 11% of patients had thrombocytopenia.

The frequencies of the clinical manifestations of the 100 SLE patients were studied. As shown in Figure 1, arthritis was the most prevalent manifestation where 77% of patients had arthritis followed by a malar rash with a percent of 70% then nephritis with a percent of 58%. Neuropsychiatric manifestations were the least prevalent manifestation where only 4% of patients developed CNS manifestations.

For TLR7 rs3853839 SNP investigation, CC genotype and C allele were selected as references. Upon Comparing CG genotype of TLR7-rs3853839 to the wild genotype CC, significant difference was found (P value = 0.04, OR 0.53, CI 95% 0.30 to 0.98) also, the G-allele of TLR7-rs3853839 was associated with SLE occurrence in our study (G/C, P = 0.01, OR 0.55, 95% CI 0.34 to 0.89). Taking GG genotype and G allele as a reference, no significant differences were found between TLR9 rs352140 (alleles and genotypes) in all studied SLE patients and healthy control subjects (Tables 1 and 2). The P-value was >0.05. None of the examined SNPs significantly deviated from Hardy Weinberg Equilibrium in the control group.

**Table 1:** Distribution of TLR7 rs3853839 and TLR9 rs187084 Genotypes in SLE patients and healthy control.

Gene polymorphism	Genotypes	Control N = 100		SLE N = 100		OR	95% CI	P-value
		N	%	N	%			
**TLR7 rs3853839**	**CC**	70	70	54	54	Reference		
	**CG**	27	27	39	39	0.53	0.30 to 0.98	0.040∗
	**GG**	3	3	7	7	0.33	0.08 to 1.34	0.100ns
**TLR9 rs187084**	**TT**	32	32	46	46	Reference		
	**TC**	41	41	33	33	1.79	0.94 to 3.40	0.080ns
	**CC**	18	18	21	21	1.23	0.57 to 2.67	0.600ns

∗, ∗∗, ∗∗∗, significant at *p* < 0.05, <0.01, <0.001; ns, non-significant at *p* > 0.05; OR odds ratio, CI confidence interval.

**Table 2:** Comparison between controls and the SLE patients regarding TLR7 rs3853839 and TLR9 rs187084 alleles.

Gene polymorphism	Alleles	Control N	%	SLE N	%	OR	95% CI	P-value	
**TLR7 rs3853839**	**C**	167	83.5	147	73.5	Reference			
	**G**	33	16.5	53	26.5	0.55	0.34 to 0.89		0.010∗∗
**TLR9 rs187084**	**T**	105	52.5	125	62.5	Reference			
	**C**	77	38.5	75	37.5	1.22	0.81 to 1.84		0.340ns

∗, ∗∗, ∗∗∗, significant at *p* < 0.05, <0.01, <0.001; ns, non-significant at *p* > 0.05; OR odds ratio, CI confidence interval.

We studied the association between TLR7 rs3853839 and TLR9 rs187084 genotypes and alleles with different clinical characteristics of SLE as shown in (Table 3). In our work, all genotypes and alleles TLR7-rs3853839 were associated with nephritis (CC vs CG P < 0.0001, OR 0.13, 95% CI 0.05–0.35/CC vs GG = 0.04, OR 0.10, 95% CI 0.01–0.87/CC vs CG + GG, P < 0.0001, OR 0.12, 95% CI 0.05–0.32/C vs G P = 0.0001, OR 0.21, 95% CI 0.10–0.47). We found that TLR7-rs3853839 genotype GG was associated with arthritis (CC vs GG, P = 0.01, OR 0.10, 95% CI 0.02 to 0.60).

**Table 3:** Comparison between TLR7 rs3853839 genotypes and alleles concerning clinical characteristics of the SLE study population.

Gene/rs	p	q	Clinical sign never presents			Clinical sign ever-presents			pp/pq OR (95% CI), P	pp/qq OR (95% CI), P	(pp vs. pq/qq) OR (95% CI), P	Allele test (p vs. q) OR (95% CI), P
			pp	pq	qq	pp	pq	qq				
Alopecia												
TLR7 rs3853839	C	G	39	28	7	15	11	0	0.98 (0.39–2.45), 0.96	0.17 (0.01–3.16), 0.24	1.22 (0.50–3.02), 0.66	1.48 (0.69–3.14), 0.31
TLR9 rs187084	T	C	35	25	14	11	8	7	0.98 (0.35–2.79), 0.97	0.63 (0.20–1.95), 0.42	0.87 (0.33–2.01), 0.66	0.76 (0.40–1.45), 0.41
Anemia												
TLR7 rs3853839	C	G	39	21	5	15	18	2	0.45 (0.19–1.07), 0.07	0.96 (0.17–5.50), 0.96	0.50 (0.22–1.15), 0.10	0.68 (0.36–1.30), 0.25
TLR9 rs187084	T	C	33	20	12	13	13	13	0.61 (0.24–1.57), 0.30	0.53 (0.18–1.54), 0.24	0.57 (0.25–1.33), 0.19	0.64 (0.36–1.17), 0.15
Arthritis												
TLR7 rs3853839	C	G	11	7	5	43	32	2	0.86 (0.30–2.45), 0.77	**0.10 (0.02 to 0.60), 0.01**	1.38 (0.54–3.51), 0.50	1.92 (0.95–3.89), 0.07
TLR9 rs187084	T	C	8	11	4	38	22	17	2.38 (0.83–6.80) 0.11	1.12 (0.30–4.22), 0.87	1.83 (0.69–4.81), 0.22	1.23 (0.63–2.41), 0.54
Discoid rash												
TLR7 rs3853839	C	G	49	33	5	5	6	2	0.56 (0.16–1.99), 0.37	0.26 (0.04–1.67), 0.15	0.49 (0.15–1.60), 0.24	0.53 (0.22–1.24), 0.14
TLR9 rs187084	T	C	40	28	19	6	5	2	0.84 (0.23–3.03), 0.79	1.43 (0.26–7.73), 0.68	1.01 (0.31–3.24), 0.99	1.15 (0.49–2.74), 0.75
Leukopenia												
TLR7 rs3853839	C	G	36	24	4	18	15	3	0.80 (0.34–1.89), 0.61	0.67 (0.13–3.30), 0.62	0.78 (0.34–1.76), 0.55	0.81 (0.42–1.55), 0.52
TLR9 rs187084	T	C	33	19	12	13	14	9	0.54 (0.21–1.24), 0.14	0.53 (0.18–1.54), 0.17	0.53 (0.23–1.23), 0.14	0.63 (0.35–1.14), 0.13
Malar rash												
TLR7 rs3853839	C	G	18	9	3	36	30	4	0.60 (0.24–1.53), 0.29	1.50 (0.30–7.43), 0.62	0.71 (0.30–1.68), 0.43	0.90 (0.45–1.79), 0.75
TLR9 rs187084	T	C	10	14	6	36	19	15	**2.65 (0.99 to 7.09),0.05**	1.44 (0.44–4.68), 0.54	2.12 (0.87–5.17), 0.10	1.42 (0.77–2.63), 0.27
Nephritis												
TLR7 rs3853839	C	G	34	7	1	20	32	6	**0.13 (0.05 to 0.35), <0.0001**	**0.10 (0.01 to 0.87), 0.04**	**0.12 (0.05 to 0.32), <0.0001**	**0.21 (0.10 to 0.47), 0.0001**
TLR9 rs187084	T	C	20	10	12	26	23	9	0.57 (0.22–1.45), 0.24	1.73 (0.61–4.92), 0.30	0.89 (0.40–1.98), 0.78	1.24 (0.70–2.22), 0.46
CNS symptoms												
TLR7 rs3853839	C	G	53	37	6	1	2	1	0.35 (0.03–3.99), 0.40	0.11 (0.01–2.05), 0.14	0.27 (0.03–2.69), 0.27	0.34 (0.08–1.42), 0.14
TLR9 rs187084	T	C	45	32	19	1	1	2	0.71 (0.04–11.80), 0.81	0.21 (0.02–2.47), 0.22	0.38 (0.04–3.76), 0.41	0.34 (0.08–1.48), 0.15
Oral ulcers												
TLR7 rs3853839	C	G	48	26	7	6	13	0	**0.25 (0.09 to 0.74), 0.01**	2.01 (0.10–39.48), 0.65	**3.15 (1.09 to 9.13), 0.04**	0.63 (0.30–1.35), 0.23
TLR9 rs187084	T	C	38	26	17	8	7	4	0.78 (0.25–2.42), 0.67	0.90 (0.24–3.38), 0.87	0.82 (0.30–2.26), 0.71	0.90 (0.44–1.86), 0.78
Pericarditis												
TLR7 rs3853839	C	G	49	34	7	5	0	5	0.69 (0.19–2.58), 0.59	1.67 (0.08–33.32), 0.74	0.84 (0.23–3.09), 0.79	1.07 (0.37–3.09), 0.91
TLR9 rs187084	T	C	41	30	19	5	3	2	1.22 (0.27–5.50), 0.80	1.16 (0.21–6.52), 0.87	1.20 (0.32–4.42), 0.79	1.13 (0.43–2.97), 0.81
Photosensitivity												
TLR7 rs3853839	C	G	36	20	3	18	19	4	0.53 (0.23–1.23), 0.14	0.38 (0.08–1.86), 0.23	0.50 (0.22–1.12), 0.09	0.58 (0.31–1.09), 0.09
TLR9 rs187084	T	C	24	21	14	22	12	7	1.60 (0.64–4.01), 0.31	1.83 (0.63–5.38), 0.27	1.69 (0.76–3.77), 0.20	1.53 (0.85–2.77), 0.16
Pleurisy												
TLR7 rs3853839	C	G	45	29	5	9	10	2	0.58 (0.21–1.60), 0.29	0.50 (0.08–2.99), 0.45	0.57 (0.21–1.50), 0.25	0.66 (0.31–1.37), 0.26
TLR9 rs187084	T	C	36	29	14	10	4	7	2.01 (0.57–7.09), 0.28	2.01 (0.57–7.09) 0.28	1.09 (0.41–2.85), 0.87	0.75 (0.38–1.50), 0.42
Thrombocytopenia												
TLR7 rs3853839	C	G	50	34	5	4	5	2	0.54 (0.14–2.17), 0.39	0.11 (0.02–0.65), 0.10	0.45 (0.12–1.63), 0.22	**0.04 (0.01 to 0.16),<0.0001**
TLR9 rs187084	T	C	42	29	18	4	4	3	0.69 (0.16–2.99), 0.62	0.57 (0.12–2.82), 0.49	0.64 (0.18–2.34), 0.50	0.69 (0.28–1.69), 0.42

∗, ∗∗, ∗∗∗, significant at *p* < 0.05, <0.01, <0.001; ns, non-significant at *p* > 0.05; OR odds ratio, CI confidence interval.

Also, TLR7-rs3853839 genotype CG and wild model were associated with oral ulcer (CC vs CG, P = 0.01, OR 0.25, 95% CI 0.09–0.74 and CC vs CG + GG, P = 0.04, OR 3.15, 95% CI 1.09–9.13).

Another significant association was detected, where TLR7-rs3853839 G allele was associated with Thrombocytopenia (C vs. G, P < 0.0001 OR 0.04, 95% CI 0.01–0.16).

No significant association was detected between TLR7-rs3853839 and alopecia, anemia, discoid rash, leukopenia, malar rash, CNS symptoms, pericarditis, photosensitivity, pleurisy.

Regarding TLR9 rs187084, we detected one significant association between genotype TC and malar rash, where (p = 0.05, OR 2.65, 95% 0.99–7.09), no significant associations were detected with other clinical characteristics.

## 4 Discussion

This work aimed to inspect the association between TLR7 rs3853839 and TLR9 rs187084 SNPs and SLE risk in Egyptian patients. The relation between the clinical data and the studied polymorphism was also investigated in our work.

The recent study showed that 26% of cases had alopecia, 40% had anemia, 77% had arthritis, 13% had a discoid rash, 36% had leukopenia, 70% had a malar rash, 58% had nephritis, 4% had neuropsychiatric manifestations, 19% had oral ulcers, 10% had pericarditis, 41% had photosensitivity 21% had pleurisy, and 11 had thrombocytopenia.

A new study performed by (Afifi et al., 2021) revealed that 44% of SLE patients suffered from arthritis/arthralgia followed by fever (39%). They claimed that the most common clinical manifestation was alopecia 76.1% and nephritis 65.7%. Cardiovascular damage was detected in (24.3%) of cases. Another study performed by (Elloumi et al., 2017) revealed that malar rash was detected in 30% of the cases, photosensitivity in 32%, arthritis in 12%, anemia in 55%, pleurisy in 9%, and lupus nephritis in 37% of the patients. Those different results may arise from various ethnic factors, differences in sample size, or the different disease duration of the studied patients.

In our work, the association analysis and distribution of the frequencies of TLR7 rs3853839 and TLR9 rs187084 were examined in SLE patients and the control population. For TLR7 rs3853839, there was a statistically significant association between genotype CG & CG + GG with SLE. We also found a statistically significant association between the G-allele of TLR7 rs3853839 and SLE occurrence in our studied cohort.

Our findings matched those of (Raafat et al., 2018), who found a statistically significant difference in TLR7 rs3853839 genotypes between controls and SLE patients, where SLE patients had a greater frequency of polymorphism genotypes (CG and GG) (60%) than healthy controls (34%).

Also (Enevold et al., 2014), confirmed an association between TLR7 rs3853839 polymorphism and SLE in a Danish patient. Also, following our results, a large multiethnic and multicentered study including Korean, Chinese, and Japanese participants recognized such SNP of TLR7 rs3853839 G/C as a risk factor for SLE (Raafat et al., 2018). This finding is consistent with (Elloumi et al., 2021) study in Tunisia, which claimed that males who carry the rs3853839 polymorphism had a greater chance of getting SLE.

TLR7 rs3853839 SNPs have also been linked to a higher risk of SLE in African Americans, Amerindian and European Americans,/Hispanics (Deng et al., 2013). Eastern Asians (Shen et al., 2010) and Egyptians (Raafat et al., 2018).

Different studies have reported conflicting results regarding the association between TLR9 gene polymorphisms and the risk of developing SLE.

Our study is considered the first to investigate the relation between TLR9 rs187084 SNP and SLE in the Egyptian population. Our results showed no significant association between different genotypes and alleles with SLE risk, where the p-value was >0.05).

Our results were in accordance with a recent study on Mexican patients that revealed that TLR9 polymorphisms were not associated with SLE. They reported that some TLR9 SNPs are considered risk factors for SLE development. However, not all populations have replicated similar findings (Aranda-Uribe et al., 2021).

Our results were consistent with (Hur et al., 2005; Enevold et al., 2014; Elloumi et al., 2017), who found no significant association between TLR9 rs187084 gene polymorphisms and SLE. TLR9 rs187084 (T/C) was found to have no association with SLE in Hong Kong research that included 799 Hong Kong Chinese healthy blood donors and 467 SLE patients (Ng et al., 2005).

In contrast with our result (Wang et al., 2016), reported that TLR9 rs187084 SNPs might elevate SLE risk in Asians. Also (Huang et al., 2012), examined TLR9 rs187084 polymorphism in SLE patients, and statistically significant differences have been found between the SLE patients and the control population.

This difference in the results between different studies may be due to differences from one population to another according to genetic or ethnic factors.

Our study found a significant association between the genotypic distribution of TLR7 rs3853839 according to SLEDAI, while no significant association was detected regarding age. Also, there was no significant association between the genotypic and allelic distribution of TLR9 rs187084 according to age, SLEDAI, and duration in all studied cases.

In accordance with our study (Wang et al., 2019), stated that TLR7 rs3853839 C/G SNP was associated with more severe disease (high SLEDAI score where P value = 0.02); they claimed that this association was a result of higher TLR7 expression in SLE patients caused by an upregulation of IFN-responsive genes which this SNP stimulated.

Also (Raafat et al., 2018), found no significant association between TLR7 rs3853839 and age nor SLEDAI. To our knowledge, those associations were not discussed in other studies.

The present work also showed a strong association between TLR7 rs3853839 genotypes and alleles with nephritis. Genotypes were associated with arthritis, oral ulcers, and thrombocytopenia. In contrast, no association was found with the other SLE clinical manifestations, including alopecia, anemia, discoid rash, leukopenia, malar rash, neuropsychiatric manifestations, pericarditis, photosensitivity, and pleurisy.

These results matched that of (Raafat et al., 2018), who investigated the role of the TLR7 rs3853839 polymorphism in developing several clinical signs of SLE. Except for nephritis, no statistically significant difference was found between the wild genotype carrying groups and those who take the polymorphic genotypes. TLR7 SNP rs3853839 has also been linked to lupus nephritis, according to (Kawasaki et al., 2011; Enevold et al., 2014). Also, TLR7 rs3853839 G risk allele was related to numerous clinical signs of SLE, including arthritis, malar rash, oral ulcer, photosensitivity, Thrombocytopenia, and pericardial effusion, according to (Wang et al., 2014).

In our work regarding TLR9 rs187084, the only significant association was detected with a malar rash. Also (Enevold et al., 2014), found that rs187084 SNP of TLR9 was only associated with malar rash, while (Huang et al., 2012) did not report any relationship between the TLR9 (rs187084) gene and any of SLE clinical characteristics in Chinese patients.

## 5 Conclusion

In conclusion, the present study confirmed the association between the TLR7 rs3853839 SNP and SLE in an Egyptian cohort. A strong association was found between rs3853839 and nephritis in SLE patients. Also, different TLR7 rs3853839 genotypes and alleles were associated with other clinical manifestations such as arthritis, oral ulcer, and Thrombocytopenia.

Our results advocate those genetic variations of TLR7 rs3853839 may be considered biomarkers for the expectation of SLE phenotypes and may help develop new therapeutic interventions that may prevent the clinical complications of SLE, especially nephritis.

The role of TLR 9 polymorphism as one of the possible risk factors for SLE cannot be eliminated, and other SNPs should be investigated.

### 5.1 Study limitations

The reduced sample size might be a limitation to our study, we also have been limited by financial constraints as it is self-funded study. It is highly recommended to analyze the correlation of TLR9 SNP with anti-dsDNA/ANA/anti-RO/La auoAbs, as well as the correlation between TLR7/TLR9 SNP and TLR7/TLR9 mRNA expression.

## Declarations

### Author contribution statement

Marwa M. Azab: Conceived and designed the experiments; Analyzed and interpreted the data; Contributed reagents, materials, analysis tools or data; Wrote the paper.

Fatma M. Mostafa: Conceived and designed the experiments; Performed the experiments; Analyzed and interpreted the data; Contributed reagents, materials, analysis tools or data; Wrote the paper.

Mayada Khalil; Mona Salama: Analyzed and interpreted the data; Contributed reagents, materials, analysis tools or data.

Ali A. Abdelrahman: Conceived and designed the experiments; Analyzed and interpreted the data; Contributed reagents, materials, analysis tools or data.

Aya A. Ali: Analyzed and interpreted the data; Contributed reagents, materials, analysis tools or data; Wrote the paper.

### Funding statement

This research did not receive any specific grant from funding agencies in the public, commercial, or not-for-profit sectors.

### Data availability statement

Data included in article/supp. material/referenced in article.

### Declaration of interest's statement

The authors declare no conflict of interest.

### Additional information

No additional information is available for this paper.
